# Estimation of the incidence of genital warts and the cost of illness in Germany: A cross-sectional study

**DOI:** 10.1186/1471-2334-8-76

**Published:** 2008-06-02

**Authors:** Peter Hillemanns, J Gabrielle Breugelmans, Friederike Gieseking, Stève Bénard, Emilie Lamure, Kavi J Littlewood, Karl U Petry

**Affiliations:** 1 Klinik für Frauenheilkunde und Geburtshilfe, Medizinische Hochschule Hannover, Hannover, Germany; 2 Sanofi Pasteur MSD, Lyon, France; 3 Universitätsklinikum Hamburg, Germany; 4 st[è]ve consultants, 14 rue Grenette, 69002, Lyon, France; 5 Mapi Values, Houten, The Netherlands; 6 Frauenklinik im Klinikum der Stadt Wolfsburg, Wolfsburg, Germany; 7 Agence de Médecine Préventive, Paris, France; 8 IMS Health, 92800, Puteaux, France

## Abstract

**Background:**

Human papillomavirus (HPV) is a necessary cause of cervical cancer. HPV is also responsible for benign *condylomata acuminata*, also known as genital warts. We assessed the incidence of genital warts in Germany and collected information on their management to estimate the annual cost of disease.

**Methods:**

This was a multi-centre observational (cross-sectional) study of genital warts in Germany. Data were collected from gynecologists, dermatologists, and urologists seeing patients with genital warts between February and April 2005. The number of patients with new and recurrent genital warts was used to estimate the incidence in Germany. We assessed resource use for patients with genital warts seen during a two-month period as well as retrospective resource use twelve months prior to the inclusion visit through a chart review. The mean costs of treatment of patients with genital warts from third-party payer and societal perspectives were estimated, and the total annual cost of genital warts was then calculated.

**Results:**

For the incidence calculation 217 specialists provided information on 848 patients and 214 specialists provided resource use data for 617 patients to assess resource consumption. The incidence of new and recurrent cases of genital warts was 113.7 and 34.7 per 100 000, respectively, for women aged 14–65 years consulting gynecologists. The highest incidence was observed in women aged 14–25 years (171.0 per 100 000) for new cases and in women aged 26–45 years (53.1 per 100 000) for recurrent cases. The sample size for males was too small to allow a meaningful estimate of the incidence. The mean direct cost per patient with new genital warts was estimated at 378 euros (95% CI: 310.8–444.9); for recurrent genital warts at 603 euros (95% CI: 436.5–814.5), and for resistant genital warts at 1,142 euros (95% CI: 639.6–1752.3). The overall cost to third-party payers was estimated at 49.0 million euros, and the total societal cost at 54.1 million euros, corresponding to an average cost per patient of 550 euros and 607 euros, respectively.

**Conclusion:**

The societal burden and costs of managing and treating genital warts in Germany are considerable. A vaccination programme using the quadrivalent human papillomavirus vaccine could provide a substantial health benefit and reduce the costs associated with genital warts in Germany.

## Background

Human papillomavirus (HPV) is a necessary cause of cervical cancer [1-3]. HPV is also responsible for benign *condylomata acuminata*, also known as genital warts. It has been estimated that as many as 75% of the sexually active population (15–49 years of age) in the US have been infected with HPV during their lifetime [4]. However, most HPV-infected individuals eliminate the virus without developing clinical symptoms [5].

To date, nearly 100 HPV types have been molecularly identified and about 40 of these can infect the anogenital tract [5-7]. On the basis of their oncogenic potential, most of these genital HPV types have been classified as high or low risk for causing cervical cancer. The high-risk types, especially HPV 16 and 18, are implicated in the development of cervical intraepithelial neoplasia and cervical carcinoma. The low-risk types (HPV 6, 11, 40, 42, 43, 44, 54, 61, 70, 72, 81, and CP6108) can cause mild cervical dysplasia but are rarely associated with severe cervical dysplasia or cervical carcinoma.

It has been reported that low-risk HPV types 6 and 11 are found in more than 90% of genital warts [8]. Clinically apparent genital warts affect about 1% (approximately 1.4 million) of the sexually active population (15–49 years) in the United States [4]. In 2003, new cases of genital warts accounted for 10% of all diagnoses at genitourinary medicine clinics in the UK [9]. Another study in the United Kingdom showed that diagnoses of new genital warts rose by 4.2% between 2003 and 2004 with the highest incidence of genital warts observed in men aged 20–24 years (783 per 100 000) and in women aged 16–19 years (703 per 100 000) [10].

Genital warts are disfiguring and can have psychosexual sequelae [11]. Treatment for genital warts includes ablative techniques or topical cytotoxic agents applied by the patients themselves or their physicians [12]. These treatments have variable response rates (60% to 90%) and can cause adverse side-effects [12].

The viral origin of genital warts means that this is a potential target for prophylactic HPV vaccines. Gardasil^®^, a quadrivalent HPV vaccine against types 6, 11, 16 and 18, was approved in 2006 by the European Medicines Agency as well as by the US Food and Drug Administration for the prevention of high-grade dysplasia of the cervix or vulva, cancer of the cervix, and genital warts [13]. The product license has been granted in many countries, such as US, Canada, Australia, Mexico, many European countries, including Germany, and the vaccine will be available soon in other countries.

Before implementing a HPV vaccination programme, it is important to understand the burden of illness, including the morbidity and costs of managing and treating patients with HPV-associated diseases. Only sparse data are currently available in Germany for the burden of illness caused by genital warts. We, therefore, performed a study to assess the incidence of genital warts, to gather information on their management, and to estimate the annual cost of genital warts in Germany.

## Methods

The study was composed of two parts; an epidemiological survey including aggregated data on genital warts cases to estimate the incidence of genital warts in Germany and a chart review to estimate the annual cost for treating genital warts.

### Estimation of the incidence of genital warts

To assess the incidence of genital warts it was estimated that data from 600 patients would provide a 95% confidence interval (95% CI) of 0.02% to 1.8%, based on an expected prevalence of 1%. Assuming that urologists, gynecologists, and dermatologists see on average 10–15, 1–3, and 12 patients with genital warts per month, respectively, (personal communication, Market Research Agency, Germany) the following sample size of investigators was required to obtain data on at least 600 patients: 130 gynecologists, 50 dermatologists, and 20 urologists. To obtain a representative geographical distribution of these specialists, data from the eight AC Nielsen regions of Germany were used to calculate the appropriate number of specialists to be recruited from each region [14]. Physicians participating in this part of the study were asked to complete a questionnaire to provide information on (i) the size of their medical practice (total number of patients seen in the previous year), (ii) the number of patients aged 14–65 years diagnosed with genital warts in the previous year, (iii) the average number of patients with genital warts seen per month, and (iv) the distribution of men and women aged 14–65 years among patients diagnosed with genital warts in the previous year. They were also asked to record information on the patients who consulted them for genital warts between 9 February and 6 April 2005. These patients were classified as new (incident) cases (diagnosed at the time of the visit) or existing (previously diagnosed) cases. When information was available, the latter were further classified as recurrent (those with previous episodes of genital warts that had resolved) or resistant (those who had previous episodes of genital warts that had not resolved with treatment). Since the study did not include any personal identifiers or direct patient involvement, ethical approval was not necessary.

The total number of cases of genital warts in Germany was extrapolated using the number of patients with genital warts per specialist per case type. Case type was defined as new or existing genital warts with the latter including recurrent or resistant genital warts. When no information was available in the medical records to classify the genital warts as recurrent or resistant, they were classified as unknown type. The age-specific incidence and incidence in the at-risk population (14–65 years) were calculated for women consulting gynecologists. The number of men included was small and therefore it was not possible to calculate the incidence of genital warts for men. The total and age-stratified population data were obtained from Eurostat [14,15].

### Resources consumption and cost analysis

The physicians contacted in the epidemiological survey were also approached to participate in the cost analysis study. The specialists were asked to provide data for patients who consulted for genital warts between 9 February and 6 April 2005 and to record planned resource use at this inclusion visit and to extract retrospective resource use from medical records covering the twelve months prior to the inclusion visit. The goal was to collect resource use data for at least 600 patients aged between 14 and 65 years, resident in Germany, who consulted a specialist for genital warts. Data for patients with only papillomatosis or other condylomata (giant condyloma, keratotic genital warts, or condyloma plana) were not collected. The patients were classified by their case type as having new (incident) or existing genital warts (as defined above). For existing cases the data were collected both for the inclusion visit and from their medical records for the 12 months prior to the inclusion visit. The data included demographic and clinical data, information on resource use (specialists visits, diagnostics, medication, procedures, adverse effects, and hospitalization), and days of sick leave. For new patients, only data obtained during the inclusion visit were included as there was no prospective data collection in this study and there was only a 2-month inclusion period.

#### Unit costs for resources

Average unit costs for consultations, diagnostic tests and treatments (drugs and procedures) related to genital warts in Germany for 2005 were compiled from the "Einheitlicher Bewertungsmassstab" (EBM), the "Gelbe Liste", the "Gebührenordnung für Ärzte", and the German Diagnosis Related Groups (Table 1) [16-19]. The cost arising from loss of work days was based on the gross domestic product (GDP), the population in Germany in 2004, and the number of days worked per year (Table 1) [15,20,21].

**Table 1:** Unit cost for resources used in the management and treatment of genital warts

	**Resource**	**Unit cost (euros)**
**Outpatient visits**^a^	Basic visit	19.37
**Office-based treatments**^a^	Cryotherapy, electrosurgery, laser therapy	19.37
	Curettage	86.59
	Trichloroacetic acid	38.37

**Diagnostic tests**^a,b^	Acetic acid tests/solcoderman	38.37
	Anoscopy/proctoscopy	8.86
	Biopsy	86.59
	Colposcopy	4.25
	Histological examination	26.34
	Hybrid capture II (HPV DNA test)	40.00
	Pap smear	5.42
	Polymerase chain reaction (HPV PCR)	16.40
	Urethroscopy/meatoscopy (men)	47.46
	Urethroscopy/meatoscopy (women)	29.44
	Visual examination	0

**Hospital-based treatments**^c^	Colposcopy	1,490.60
	Curettage, biopsy	1,252.80
	Electosurgery or laser therapy (men)	1,403.60
	Electosurgery or laser therapy (women)	1,464.50
	Proctoscopy (men)	1,403.60
	Proctoscopy (women)	1,490.60
	Urethroscopy	1,403.60

**Medications **^d^	Aldara^® ^5% cream	99.78
	Condylox^® ^solution	35.26
	Solcoderman	38.37
	Triapten (2 g)	16.91
	Triapten (6 g)	45.00
	Virudermin gel	4.85
	Wartec^® ^cream 0.15%	39.50

**Indirect cost**	Lost work day (GDP/person/day)^e^	118.89

^a^Einheitliche Bewertungsmasstab (EBM) [16]. ^b^Gebührenordnung für Ärzte (GOA) [17]. ^c^German Diagnosis Related Groups [18].^d^Gelbe Liste Pharmindex [19]. ^e^Calculated as: Gross domestic product (GDP) for Germany in 2004 (2207 billion euros)/population in 2004 (82,500,705; and number of working days per year (225) [15, 20, 21].

#### Statistical analysis of the healthcare cost data

The cost per patient was calculated by multiplying the units of resources used by the unit costs. The mean cost per patient for each genital wart type was calculated and the 95% confidence intervals (95% CI) were obtained using non-parametric bootstrapping techniques because the distribution of costs was expected to be skewed. The impact of patient characteristics (type of patient, type of wart, gender, infection status and age) and type of specialist on the overall costs were evaluated in a multivariable analysis. An F-test was performed to determine the overall significance of the model. The significance of each parameter in the model was determined using a *t*-test. Statistical analyses were conducted using SAS version 8 (SAS Institute, Inc.).

#### Estimated total annual cost of genital warts in Germany

The cost of illness for each type of genital warts in Germany was calculated by multiplying the estimated total number of patients per year by the mean annual cost per patient for each type. The costs for existing cases of unknown type were estimated using the weighted average costs for recurrent and resistant cases based on the observed proportions. The total annual cost of genital warts in Germany was then calculated as the sum of the costs for the four types. The analysis was performed from both the third-party payer (direct medical cost only) and societal (direct medical cost and cost of lost productivity) perspectives. The cost for specialist visits, diagnostic tests, medication, and procedures were included in the direct medical costs, and the cost of lost work days was considered in the indirect cost. The cost for retrospective and current resource use was included in the costs for patients with resistant and recurrent lesions. For patients with new genital warts, the costs were based on the current visit and the prescriptions by the physician during the consultation. The overall cost to third-party payers and the total societal cost included both women and men, and all specialists.

## Results

### Incidence of genital warts

Between January and March 2005, 401 specialists were invited by telephone to participate in the epidemiological survey on genital warts. Of these, 217 agreed to participate: 135 gynecologists, 55 dermatologists, and 27 urologists. Data for 848 patients with genital warts were collected for the study, including 508 new patients (incident) and 340 patients with existing genital warts (168 recurrent, 42 resistant, and 130 status unknown) (Table 2A). The majority of the patients were women (71%) and most (86%) consulted a gynecologist for their genital warts. Men consulted a dermatologist (61%) more often than an urologist for genital warts (39%). Table 2B shows the age-specific incidence of genital warts in women consulting gynecologists. The overall incidence for new cases was 113.7 (95% CI: 108.6–118.7) per 100 000 for women aged 14–65 and 76.0 (95% CI: 72.6–79.4) per 100 000 of the total female population. The overall incidence for recurrent cases was 34.7 (95% CI: 32.1–37.4) per 100 000 for women aged 14–65 and 23.2 (95% CI: 21.4–25.0) per 100 000 of the total female population. The highest age stratified incidence for new cases was observed in women aged 14–25 years (171.0 [95% CI: 163.4–178.5] per 100 000) while for recurrent cases it was higher in women aged 26–45 years (53.1 [95% CI: 49.0–57.2] per 100 000). The number of males visiting dermatologists or urologists was too limited to provide a meaningful incidence of genital warts in this group. However, the observed numbers of men with genital warts during the study period were used in the extrapolation to approximate the total number of cases (male and female) of genital warts seen by physicians in Germany (Table 3). Using the observed numbers (Table 2A) we estimated that there were 54,358 new, 16,520 recurrent, 3982 resistant, and 14,193 existing (status unknown) cases of genital warts in Germany, annually (Table 3).

**Table 2:** 

A. Observed numbers of men and women with genital warts seen annually by physicians included in the study										
	**New**		**Recurrent**		**Resistant**		**Unknown (existing)**		**Total**	
					
	**Men**	**Women**	**Men**	**Women**	**Men**	**Women**	**Men**	**Women**	**Men**	**Women**

Gynecologists	0	303	0	104	0	29	0	95	0	531
Dermatologists	87	55	29	18	7	5	16	11	139	89
Urologists	63	0	17	0	1	0	8	0	89	0

**Sub-total**	**150**	**358**	**46**	**122**	**8**	**34**	**24**	**106**	**228**	**620**
**Total**	**508**		**168**		**42**		**130**		**848**	


B. Age-specific incidence of genital warts in women consulting gynecologists										

	**Incidence per 100 000 (95% CI)**									
	
**Age (years)**	**New genital warts**					**Recurrent genital warts**				

14–25	171.0 (163.4–178.5)					52.8 (48.7–56.8)				
26–45	156.5 (149.5–163.4)					53.1 (49.0–57.2)				
46–65	36.3 (34.7–37.9)					5.0 (4.6–5.3)				
14–65^a^	113.7 (108.6–118.7)					34.7 (32.1–37.4)				
All ages	76.0 (72.6–79.4)					23.2 (21.4–25.0)				

^a^The total at-risk population (14–65 years of age).

**Table 3:** Estimated numbers of men and women with genital warts seen annually by physicians in Germany

	**New cases (95% CI)**		**Recurrent cases (95% CI)**		**Resistant cases (95% CI)**		**Existing cases (unknown type) (95% CI)**	
				
	**Men**	**Women**	**Men**	**Women**	**Men**	**Women**	**Men**	**Women**
**Gynecologists**	-	31,958 (29,180–34,738)	-	9,768 (8,291–11,245)	-	3,013 (2,102–3,925)	-	10,812 (8,981–12,643)
**Dermatologists**	7,721 (7,050–8,392)	4,937 (3,962–4,716)	2,454 (2,082–2,825)	1,569 (1,331–1,806)	556 (388–724)	355 (248–463)	1,430 (1,188–1,672)	914 (759–1,069)
**Urologists**	9,742 (8,895–10,589)	-	2,729 (2,116–3,142)	-	58 (40–75)	-	1,037 (862–1,213)	-

**Total**	**17,463 (15,944–18,982)**	**36,895 (33,687–40,103)**	**5,183 (4398–5966)**	**11,337 (9622–13,050)**	**614 (428–780)**	**3,368 (2,350–4,388)**	**2,467 (2,049–2,887)**	**11,726 (9,741–13,712)**

### Resources use and cost analysis

A total of 214 physicians participated in the chart review study, including 129 gynecologists, 59 dermatologists, and 26 urologists. More than 90% of the specialists were office-based, and the others were based in hospitals. Most of the specialists (85%) worked in their own medical practice. Data for 621 patients were collected; data for four of these patients were excluded because they did not meet the age criteria (n = 3) or had a treatment duration of over 12 months (n = 1).

#### Patient characteristics

The study sample (n = 617 patients) included 384 women (62%) and 233 men (38%). The mean age was 32.0 ± 10.0 years. There was no significant difference in age between the patients with new (32.2 ± 10.1 years) and existing (31.6 ± 9.9 years) genital warts. Treatment follow-up time was not available for 12 of the 189 patients with existing genital warts. The remaining 177 patients had a mean follow-up of 6.7 ± 4.3 months. Approximately half (48%) of the patients in the study had an elementary or primary school education, 28% had a secondary or high school education, and 12% had a college or postgraduate degree. Of the 352 patients with data available, the majority (95%) reported having had three or fewer new sexual partners in the previous year: 13.9% had no new partner, 52.0% had one new partner, 20.7% had two new partners and 7.9% had three new partners and 5.5% had more than three new partners. Most of the patients (76%) reported that they had not had a sexually transmitted infection previously.

#### Clinical data

Sixty-nine percent (n = 428) of the patients were newly diagnosed with genital warts; 15% had recurrent genital warts (n = 92), 6% had resistant genital warts (n = 36) and for 10% (n = 61) the type of existing genital warts was not specified (Table 4). Almost all the patients (95%) had acuminate warts, but due to the diagnostic similarities between the different types of warts, some physicians included patients with papular warts (4%), macular lesions (0.3%), or a combination of acuminate warts and other types of warts (0.5%). Most patients (74%) had external lesions, 21% had both internal and external lesions and 5% had only internal lesions. External lesions in women included lesions on the following anatomical sites: fourchette, labia minora and majora, clitoris, urethral meatus, perineum, anal area, vestibule, introitus (vaginal orifice), and vagina. Internal lesions were found on the ectocervix. Fifty-seven percent of all women had genital warts on the perineum, followed by the introitus (35%), labia minora (35%), anal area (35%), labia majora (34%), and vagina (21%). Only a few women were reported to have lesions on the ectocervix (8%). External lesions in males included lesions on the following sites: glans, coronal sulcus, fraenulum, foreskin, scrotum, groin, perineum, anal area, penile shaft, and meatus. Internal lesions were located in the urethra. In males, the shaft of the penis was often affected by genital warts (41%), followed by the glans (30%), the coronal sulcus (28%), foreskin (24%), anal area (21%), fraenulum (19%) and scrotum (12%). Only a few men were reported to have lesions on the groin (6%), perineum (5%) or urethra (4%).

**Table 4:** Distribution of patients included in the chart review survey

	**New cases **		**Existing cases**						
				
			**Recurrent**		**Resistant**		**Unknown type**		
					
	**Women**	**Men**	**Women**	**Men**	**Women**	**Men**	**Women**	**Men**	**Total**
Gynecologist	253	1	53	0	14	0	39	0	**360**
Dermatologist	15	102	2	25	5	17	3	17	**186**
Urologist	0	57	0	12	0	0	0	2	**71**

**Sub-total **	268	160	55	37	19	17	42	19	
**Total**	**428**		**92**		**36**		**61**		**617**

#### Resource use by the patients

Of the 428 patients consulting a specialist for new genital warts 254 (59%) saw a gynecologist, 117 (27%) a dermatologist, and 57 (13%) an urologist (Table 4). Of the 189 patients consulting for existing genital warts, 106 (56%) consulted a gynecologist, 69 (37%) a gynecologist, and 14 (7%) an urologist at least once during the one-year data collection period. The patients with existing genital warts of unknown status (n = 61) were not included in the resource use analysis. New patients had no retrospective resource use as they were included in the study on their first visit for a genital wart episode. Patients with existing genital warts had a mean follow-up of 6.71 ± 4.29 months and most had up to five consultations during this period. The mean number of visits per year was slightly higher for patients with resistant genital warts than for other types of cases (data not shown).

For patients with new genital warts (n = 428) the most frequent diagnostic tests were visual examination (36.7%), Pap smear tests (15.7%), colposcopy (12.6%) and biopsy (10.7%). For those with recurrent (n = 92) and resistant genital warts (n = 36) visual examination was by far the most frequent diagnostic test (94.6% and 97.2% respectively), followed by Pap smear tests (32.6%), and biopsy and colposcopy (26.1% for each) and 19.6% had histological tests. Imiquimod, podophyllotoxin solution, and podophyllotoxin cream were the most frequently self-administered medications for all patients (data not shown). There was no difference in the frequency of use of these therapies for new, recurrent, and resistant genital warts. Table 5 shows the distribution of physician-administered therapies for patients with new, recurrent, and resistant genital warts. The most common therapy for patients with new and recurrent lesions was electrosurgery (19.2% and 31.5%, respectively) compared with cryotherapy (33.3%) for those with resistant lesions. More than one-third of the patients, regardless of their type of lesion, did not have a procedure at the physician's office at the time of the visit. Almost 35% of patients with recurrent genital warts and 30% of those with resistant genital warts experienced adverse events after treatment. The main reported adverse events were burning, skin reactions and pain in the warts area. The percentage of patients with recurrent and resistant genital warts who were hospitalized was 10.9% and 19.4%, respectively, with a median of one hospitalization per patient (maximum 3 and 2, respectively). Patients hospitalised for resistant warts (n = 7) and for recurrent warts (n = 10) had an average length of stay of 5 days and 3 days, respectively (median 4 days and 1 day) The median number of sick leave days was similar for patients with recurrent genital warts (6 days – range 1–5) and with resistant genital warts (7 days – range 3–14), but 25% of patients with resistant genital warts took sick leave compared with 14.1% of those with recurrent warts.

**Table 5:** Frequency of use of office therapies (procedures) for genital warts

	**New genital warts (n = 428)**		**Recurrent genital warts (n = 92)**		**Resistant genital warts (n = 36)**	
			
**Procedure**	**%**	**Mean (SD)**	**%**	**Mean (SD)**	**%**	**Mean (SD)**
None	38.3		50.0		44.4	
Condylox/Phodophylotoxin Iocal/Podophlin solution	5.1	1.2 (0.6)	12.0	1.7 (1.2)	25.0	2.0 (0.5)
Cryotherapy	7.2	2.7 (2.2)	13.0	2.3 (1.4)	33.3	3.2 (3.0)
Curettage	5.1	1.2 (0.5)	9.8	1.8 (1.1)	13.9	1.6 (0.9)
Electrosurgery	19.2	1.3 (1.1)	31.5	1.7 (1.1)	22.2	2.0 (1.4)
Excision	1.4	1.0 (0.0)	5.4	1.0 (0.0)	2.8	1.0 (0.0)
Laser therapy	14.0	1.0 (0.1)	15.2	1.3 (0.5)	25.0	1.2 (0.4)
Radiation therapy	0.0	-	1.1	6.0 (0.0)	2.8	6.0 (0.0)
Solcoderman	2.8	1.3 (0.6)	1.1	4.0 (0.0)	2.8	4.0 (0.0)
Surgery	0.0	-	1.1	1.0 (0.0)	5.6	2.0 (1.4)
Trichloroacetic acid	8.2	2.9 (2.1)	9.8	2.1 (1.5)	13.9	3.2 (2.1)
Other (not specified)	1.2	1.8 (1.3)	1.1	1.0 (0.0)	2.8	3.0 (0.0)

#### Estimated mean cost per patient with genital warts in Germany

All costs were highest for resistant genital warts: 1,141.8 euros (95% CI: 639.6–1752.3) for the mean annual direct cost and 1,369.8 euros (95% CI: 830.1–1979.1) including indirect costs. Direct mean cost for recurrent genital warts were 602.7 euros (95% CI: 436.5–814.5) and 732.91 euros (95% CI: 514.6–961.9), including indirect costs and 377.6 euros (95% CI: (310.75–444.88) for new genital warts (included direct cost only). Table 6 shows the estimated mean annual direct and indirect costs for patients with new, resistant, and recurrent genital warts by gender. No significant differences in the mean direct and indirect costs were observed between men and women.

**Table 6:** Mean direct and indirect annual costs (euros) per patient with genital warts by gender

	**New cases**		**Recurrent cases**		**Resistant cases**	
			
	**Women n = 268**	**Men n = 160**	**Women n = 55**	**Men n = 37**	**Women n = 19**	**Men n = 17**
Specialist visits	19.37 (19.37–19.37)	19.37 (19.37–19.37)	85.58 (67.27–111.29)	60.95 (52.35–69.63)	96.59 (69.32–134.57)	83.13 (67.23–100.27)
Diagnostics	132.18 (79.54–193.05)	63.38 (27.12–106.48)	269.43 (76.26–527.44)	56.22 (12.19–128.34)	580.36 (64.13–1,229.11)	47.00 (15.75–85.46)
Medications	44.06 (35.23–54.25)	47.11 (36.41–59.98)	90.61 (61.44–120.09)	74.84 (39.42–113.22)	153.66 (76.79–239.57)	100.38 (26.40–193.69)
Procedures	217.93 (155.97–285.22)	185.60 (123.85–257.39)	286.22 (133.57–466.95)	242.04 (54.57–488.61)	731.95 (342.32–1,180.28)	469.09 (42.64–1,156.60)

Total direct costs*	413.55 (322.33–506.05)	315.46 (235.33–407.13)	731.84 (475.80–1,046.71)	434.05 (230.33–695.47)	1,562.55 (841.52–2,428.22)	699.60 (228.15–1,431.37)

Sick leave	-	-	142.07 (48.06–257.45)	115.57 (19.83–238.00)	231.67 (79.33–426.42)	203.87 (21.00–451.50)

**Total cost (direct and indirect)**	413.55 (322.33–506.05)	315.46 (235.33–407.13)	865.99 (575.18–1,219.90)	547.51 (283.35–874.30)	1,784.53 (988.26–2,662.98)	903.47 (325.65–1,710.29)

#### Estimated total annual cost of genital warts in Germany

The total estimated annual direct cost for new, recurrent, resistant, and existing genital warts of unknown status in Germany was 20.8 million euros, 10.0 million euros, 5.7 million euros, and 12.0 million euros, respectively. Including indirect costs the total expenditures rose to 12.7 million euros, 6.7 million euros, and 14.1 million euros for recurrent, resistant, and existing genital warts of unknown status, respectively. On the basis of these calculations, the total annual cost of genital warts in Germany was estimated at 49.0 million euros from a third-party payer perspective (indirect costs excluded) and 54.1 million euros from a societal perspective (indirect costs included), corresponding to an average cost per patient of 550 euros and 607 euros, respectively.

#### Multivariable analysis

Multivariable analyses identified the type of patient (new versus existing), type of wart, and location of wart (internal, external, or both) as factors significantly affecting the total cost of genital warts (*P *< 0.05) (Table 7). Gender, age of patient, and type of specialist did not have a significant impact on the total costs. With this model we calculated the difference in the mean costs for different types of patients, adjusted for the other cofactors. The models showed that existing patients had, on average, an additional cost of 284 euros (95% CI: 88 – 479) compared with new patients. Furthermore, the cost for patients with only external warts was, on average 300 euros (95%CI: 132 – 468) lower than that for those with internal and external warts. Lastly, patients with resistant warts spent on average 424 euros (95% CI: 64 – 785) more for the treatment of their warts.

**Table 7:** Results from the multivariable model (significant co-factors only)*

**Variables**	**Levels**	**Estimate**	**95% CI**		**P value**
Type of patients	Existing patients	284.17	88.85	479.49	0.0044
	New patients (referent)	0.0	.	.	

Type of warts	External	-300.43	-468.21	-132.64	0.0005
	Internal	238.40	-98.65	575.446	0.1653
	Internal and external (referent)	0.0	.	.	

Infection status	First infection	-33.75	-243.84	176.35	0.7525
	Recurrent infection	-37.16	-313.82	239.50	0.7920
	Resistant infection	424.13	63.77	784.50	0.0211
	Unknown (referent)	0.0			

* The analyses were adjusted for gender of the patient and type of specialist that treated the genital warts;

## Discussion

The incidence of genital warts was 171/100 000 in 14–25 year old women and 157/100 000 in 26–45 year old women. This study confirms that genital warts are very prevalent in young to mid-adult women in Germany. Furthermore, the results indicate an annual health and economic burden to the German health care system of at least 89 000 cases of genital warts and 54 million euros in associated costs.

The overall incidence of new genital warts estimated for Germany is lower than those reported in women in the United Kingdom and France [9,22]. Results in the United Kingdom showed an overall incidence of genital warts in women of 122 per 100 000 population compared with 76 per 100 000 in this study [9]. The peak incidence (reported in 16–19 year olds) was 703 per 100 000 population compared with 171 per 100 000 in 14–25 year olds in the present study. A direct comparison of these findings is difficult because of the different methodologies used in the studies. In the United Kingdom, data were obtained for one year in an ongoing well-established genital warts surveillance system, while our results were obtained through a cross-sectional study over a two-month period using a convenient sample of physicians [9]. Our estimates are also lower than those reported in a study in France that used similar methodology [22]. In this latter study with a convenient sample of 212 gynecologists the incidence reported was 176 and 48 per 100 000 females aged 15 to 65 years old for new cases and recurrent cases, respectively. Differences in HPV prevalence in Germany, France and the UK may account for some of the disparity, but we cannot rule out that the incidence of genital warts has been underestimated in the present study. A reliable estimate of the incidence of genital warts in males could not be calculated because data were not available for a sufficient number of men. However, despite the small sample size and the inherent limitations associated with small sample sizes (e.g. higher margins of error in the estimate), we used all the data collected in the epidemiological survey for the resource consumption part of the study to obtain an estimate of the total number of cases (male and female) of genital warts per year in Germany.

A report on the management of genital warts in the UK estimated that the mean direct cost per new and recurrent patient was £180 (266 euros) and £229 (338 euros), respectively [23]. In The Netherlands, the mean cost for an episode of care was 221 euros for men and 292 euros for women [24]. These costs are lower then the mean direct cost reported in the current study (550 euros). Another recent cost analysis in France found that the estimated mean annual direct costs per patient with new, recurrent, and resistant genital warts were 416 euros, 620 euros, and 970 euros, respectively [22]. These results are similar to those of the current study for new and recurrent genital warts but the cost of resistant genital warts is lower in France.

The study has several limitations. Although the physicians who participated in this study were representative in terms of geographic distribution and type of practice (90% were office-based, 85% worked in the private sector) we cannot exclude that physicians who did not participate were more or less likely to see patients with genital warts. Also some of the physicians participating in the study reported no patients consulting for genital warts during the 2-month study period and some could not provide an accurate estimate of the number of patients with genital warts in their practice. In addition, we could not verify if the patients with genital warts seen by the physicians participating in the study were representative out of all patients with genital warts in Germany. In addition, we assumed that all patients with genital warts consult a physician; however, it is possible that some patients with genital warts decided not to seek medical care because they do not consider that the condition is serious enough to seek medical care. If so, ascertainment bias may have resulted in an underestimation of the true incidence of genital warts in our study. Our results show the incidence of patients seeking medical care for genital warts. These biases may have resulted in either an over- or underestimation of the true incidence of genital warts in Germany.

A second limitation is the extrapolation of the total number of men with genital warts. The incidence of genital warts in men visiting dermatologists or urologists was not calculated because too few data on males with genital warts were available to provide a reliable estimate of the incidence. From data obtained from the genital warts surveillance system in the UK it is known that the incidence for genital warts is comparable for men (146 per 100 000 population) and women (122 per 100 000 population) [10]. Therefore, the total number of male genital warts cases in Germany may be underestimated.

Lastly, our estimate of the total cost of genital warts in Germany is likely to be underestimated. Annual resource consumption data was collected only for existing patients. For new patients, only the costs related to the first visit were included, and no indirect costs were included. Moreover, the estimated number of men with genital warts is likely to be underestimated, resulting in an underestimation of the associated cost.

Recent results from clinical trials of prophylactic vaccination of women between 16 and 24 years of age with a quadrivalent HPV (types 6,11,16,18) L1 virus-like particle vaccine (Gardasil) have shown 100% vaccine efficacy for the co-primary composite endpoints; incidence of genital warts, vulvar or vaginal intraepithelial neoplasia or cancer and incidence of cervical intraepithelial neoplasia, adenocarcinoma in situ or cancer associated with HPV type 6, 11, 16 or 18 (per-protocol susceptible population) [25]. This vaccine (Gardasil) was approved by the European Medicines Agency and the US Food and Drug Administration in 2006 for the prevention of high-grade dysplasia of the cervix or vulva, cancer of the cervix, and genital warts. Since more than 90% of genital warts cases are associated with infection by HPV types 6 and 11, our results suggest that a vaccination programme using Gardasil should reduce the medical and societal costs associated with genital warts management in Germany.

## Competing interests

JGB, SB and EL previously worked for Sanofi Pasteur MSD, who commercializes Gardasil^® ^in Europe. PH declared that he has received honorarium for lectures and clinical studies from Sanofi Pasteur MSD, GSK; Digene and Cytyc. KJL declared that she worked for MAPI Values who were contracted by Sanofi Pasteur MSD to analyse the study data. FG and KUP declared that they have received honorarium from Sanofi Pasteur MSD and GSK for speaking at several scientific meetings and for acting as scientific consultants.

## Authors' contributions

PH, JGB, FG, SB, and KUP were involved in the study conception, JGB and EL were involved in the coordination, KJL performed the data analyses, all authors were involved in the interpretation and validation of the results, PH, JGB and KUP were involved in the drafting of the manuscript and all authors read and approved the final manuscript.

## Pre-publication history

The pre-publication history for this paper can be accessed here:


